# Phloretin, an Apple Phytoalexin, Affects the Virulence and Fitness of *Pectobacterium brasiliense* by Interfering With Quorum-Sensing

**DOI:** 10.3389/fpls.2021.671807

**Published:** 2021-06-25

**Authors:** Manoj Pun, Netaly Khazanov, Ortal Galsurker, Michal Weitman, Zohar Kerem, Hanoch Senderowitz, Iris Yedidia

**Affiliations:** ^1^The Institute of Plant Sciences, Volcani Center, Agricultural Research Organization (ARO), Rishon Lezion, Israel; ^2^The Robert H. Smith Faculty of Agriculture, Food and Environment, The Hebrew University of Jerusalem, Rehovot, Israel; ^3^Department of Chemistry, Bar-Ilan University, Ramat Gan, Israel

**Keywords:** AHL synthase, N-acylhomoserine lactone, *Pectobacterium brasiliense*, phloretin, quorum sensing inhibitors, virulence

## Abstract

The effects of phloretin a phytoalexin from apple, was tested on *Pectobacterium brasiliense* (Pb1692), an emerging soft-rot pathogen of potato. Exposure of Pb1692 to 0.2 mM phloretin a concentration that does not affect growth, or to 0.4 mM a 50% growth inhibiting concentration (50% MIC), reduced motility, biofilm formation, secretion of plant cell wall-degrading enzymes, production of acyl–homoserine lactone (AHL) signaling molecules and infection, phenotypes that are associated with bacterial population density-dependent system known as quorum sensing (QS). To analyze the effect of growth inhibition on QS, the activity of ciprofloxacin, an antibiotic that impairs cell division, was compared to that of phloretin at 50% MIC. Unlike phloretin, the antibiotic hardly affected the tested phenotypes. The use of DH5α, a QS-negative *Escherichia coli* strain, transformed with an AHL synthase (ExpI) from Pb1692, allowed to validate direct inhibition of AHL production by phloretin, as demonstrated by two biosensor strains, *Chromobacterium violaceaum* (CV026) and *E. coli* (pSB401). Expression analysis of virulence-related genes revealed downregulation of QS-regulated genes (*expI*, *expR*, *luxS, rsmB*), plant cell wall degrading enzymes genes (*pel, peh* and *prt*) and motility genes (*motA, fim, fliA, flhC* and *flhD*) following exposure to both phloretin concentrations. The results support the inhibition of ExpI activity by phloretin. Docking simulations were used to predict the molecular associations between phloretin and the active site of ExpI, to suggest a likely mode of action for the compound’s inhibition of virulence.

## Introduction

Phytopathogenic pectobacteria are responsible for economically significant yield losses caused by soft rot and blackleg disease in a wide range of commercial crops, including potato (*Solanum tuberosum*), vegetables and ornamentals (Ma et al., 2007; Mansfield et al., 2012; Charkowski, 2018). *P. brasiliense* is an emerging *Pectobacterium* spp. that was first isolated in Brazil (Duarte et al., 2004), and since then has been associated with symptomatic plants throughout Europe, Africa and Asia (van der Wolf et al., 2017). The main virulence factors employed by these pathogens are the synchronized secretion of plant cell wall-degrading enzymes (PCWDEs) that promote tissue maceration and rotting by disrupting the integrity of their host cells (Liu et al., 2008; Pollumaa et al., 2012; Charkowski, 2018). The coordination of virulence factors is crucial for host infection, and is largely dependent on biotic and abiotic environmental cues such as pH, temperature, osmolarity and nutrient availability (Broberg et al., 2014). In *Pectobacteria*, a genus in the family Pectobacteriaceae (Adeolu et al., 2016), virulence regulation is under the strict control of quorum-sensing (QS), a mechanism that regulates a complex set of transcriptional factors and posttranscriptional regulators according to the level of cell density (Fuqua et al., 1994; Chatterjee et al., 1995; Koiv and Mae, 2001). This cell–cell communication system allows bacteria to sense small diffusible communication signals known as auto-inducers. The concentrations of these diffusible signaling molecules are proportional to population density and affect the regulation of gene expression in a concentration-dependent manner (Fuqua et al., 2001; Helman and Chernin, 2015). In *Pectobacterium*, QS mainly involves the production, detection and responses to the signal molecule *N*-acylhomoserine lactone (AHL), of which the most common in *Pectobacterium* are 3-oxo-C6-HSL and 3-oxo-C8-HSL (3-oxo-hexanonyl homoserine lactone and 3-oxo-octanonyl homoserine lactone, respectively) (Pollumaa et al., 2012).

The QS system in this genus includes several components that each play a central role during host infection: AHL synthase; ExpI, which synthesizes the signal molecules; and one or two response regulators, ExpR1 and ExpR2, which act as negative regulators of PCWDE production, detecting AHL signal molecules (Sjöblom et al., 2006; Pollumaa et al., 2012). In the absence of AHL, ExpR binds the promoter region of *rsmA*, thereby activating its transcription (Chatterjee et al., 2005). The Rsm system is a global post-transcriptional regulatory system mediated by RsmA-*rsmB*. RsmA is a small RNA-binding protein that is able to complex with mRNAs of PCWDE genes, to promote their degradation by RNAses. The sRNA *rsmB* binds RsmA, to form an inactive complex, thereby neutralizing its negative regulatory effect (Chatterjee et al., 2002; Pollumaa et al., 2012; Valente et al., 2017). The binding of AHL to ExpR, reduces *rsmA* expression, which, in turn, leads to the de-repression of virulence genes (Cui et al., 2005, 2006; Vadakkan et al., 2018). In a recent study, ExpI or ExpR double mutants of *P. parmentieri* were used to explore their potential as targets for QS inhibition. In the absence of ExpR, the expression of *rsmA* was downregulated, by this de-repressed virulence genes and infection. In contrast, the absence of ExpI strongly impaired virulence, supporting ExpI as a preferred target for virulence inhibition in this genus (Joshi et al., 2020). Secondary metabolites derived from plants, including phenolic acids, quinones, flavonoids, terpenoids and alkaloids, have been shown to successfully inhibit bacterial virulence (Lewis and Ausubel, 2006; Asfour, 2018). Despite their relatively mild antimicrobial effects, some compounds have been shown to be efficient QS inhibitors and represent a potential strategy for the control of bacterial soft rot (Wood et al., 2013; Joshi et al., 2016a, b, 2020; Asfour, 2018). QS proteins as potential targets for inhibition by plant-derived compounds have been shown to significantly attenuate the virulence of *Pseudomonas aeruginosa* (Hentzer et al., 2003), *Vibrio harveyi* (Manefield et al., 2000) and several *Pectobacterium* species (Manefield et al., 2001; Joshi et al., 2016a).

Many flavonoids are ubiquitous in the Plant Kingdom, biologically active and known to play roles in combating plant diseases. Phloretin is a dihydrochalcone polyphenol mainly found in apple (*Malus domestica*; about 5 mg/100 g of fresh apple), pear (*Pyrus amygdaliformis*) and strawberry fruit (Fragaria x ananassa), which acts as a natural plant-defense compound (Gitzinger et al., 2009; Lee et al., 2011). In apple, its content significantly increases in response to infection with the fire blight bacterium *Erwinia amylovora*. Therefore, it is considered a phytoalexin (Sklodowska et al., 2018). In terms of its effects on other Gram-negative bacteria, phloretin has been shown to effectively inhibit biofilm formation in *E. coli* O157:H7, but not in *E. coli* K-12 strains or in non-pathogenic *E. coli* strains, where it was found to enhance biofilm formation in a dose-dependent manner (Lee et al., 2011). It was also reported to have a significant effect on motility, reducing the swarming and swimming of *P. syringae* pv. *tomato* (Pto) strain DC3000A (Vargas et al., 2013). Phloretin, alone or in combination with citral, has also been reported to reduce biofilm formation in Gram-positive bacteria such as *Streptococcus pyogenes* (Adil et al., 2019).

The properties of phloretin were assessed in *P. brasiliense*, including its specific effects on growth, motility, biofilm formation, PCWDEs, infection, the synthesis of QS-signaling molecules, and the expression of several virulence-related genes. A possible mode of action was suggested by docking analysis of phloretin to the active binding site of ExpI, the bacterium’s AHL synthase. This is the first report on the effects of phloretin on the QS system and fitness of *P. brasiliense.* The results suggest that phloretin interferes with AHL synthesis through the inhibition of ExpI, thereby impairing bacterial virulence.

## Results

### Effect of Phloretin on Pb1692 Growth

Plants secrete an arsenal of secondary metabolites, including a vast number of flavonoids that are involved in plant defense against bacterial pathogens (Daglia, 2012; Joshi et al., 2015). To study the effect of phloretin on the growth and virulence of Pb1692, we first assessed its effect on growth and viability following exposure to increasing concentrations (0, 0.1–0.5 mM and 1.0 mM) of the phytochemical in a minimum inhibitory concentration (MIC) assay. To determine the MIC and a non-lethal concentration of phloretin that would inhibit growth by less than 50%, curves of bacterial growth in the presence of phloretin were constructed (Supplementary Figure 1A). The antibiotic ciprofloxacin that inhibits cell division was added at 50% growth inhibiting concentration (50% MIC) as an additional treatment. Bacterial cells at the end point of the experiment, were also enumerated by serial dilution plating (Supplementary Figure 1B). The lowest concentration of phloretin that completely inhibited bacterial growth was 1 mM; whereas 0.4 mM caused less than 50% growth inhibition (Supplementary Figure 1). Ciprofloxacin at a concentration of 0.013 mM (5 ng/mL) resulted in 50% growth inhibition. Based on these results and to further explore the effects of phloretin on the virulence of Pb1692, we used the following non-lethal phloretin concentrations: 0.1, 0.2, and 0.4 mM.

### Effects of Phloretin on Pb1692 Motility and Biofilm Formation

**FIGURE 1:**
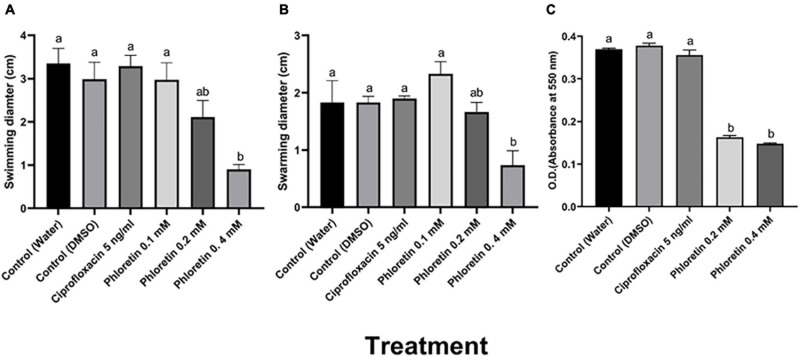
Effects of non-lethal phloretin concentrations on **(A)** swimming motility, **(B)** swarming motility and **(C)** biofilm formation *of Pectobacterium brasiliense* (Pb1692). A dH_2_O treatment and a 0.3% DMSO treatment were used as controls. Bacteria were exposed to 0.1, 0.2, 0.4 mM phloretin or ciprofloxacin, 5 ng/mL (50% growth inhibiting concentration). The motility diameter was measured after 24 h. Bars that are not labeled with same letter are significantly different from each other (*P* < 0.05; *n* = 16); Data represent the means ± SE of 2 independent experiments, each with 8 replicates. Biofilm formation was measured after 72 h of growth in LB medium at 28°C. Each bar represents the absorbance of crystal violet dye (bound to the biofilm cells) measured at 550 nm. Bars that are not labeled with the same letter are significantly different from each other (*P* < 0.05; *n* = 12). One-way ANOVA and *post hoc* Tukey-Kramer HSD tests were used to analyze differences. The analysis was performed using GraphPad Prism 8.0. Data represent means ± SE of 2 independent experiments, each with 6 replicates.

Motility is an important determinant of the virulence of plant-pathogenic bacteria including *Pectobacterium* spp. (Cui et al., 2008). To determine the effects of phloretin on motility, swimming and swarming motility of Pb1692 were assessed. The results indicated that phloretin at a concentration of 0.4 mM significantly reduced the swimming and swarming motility of Pb1692 (Figures 1A,B). The 75% reduction in swimming motility was dose dependent. In contrast, ciprofloxacin did not affect motility under the same growth inhibiting concentrations (Figures 1A,B).

The survival and persistence of plant-pathogenic bacteria depends, to a large extent, on their ability to colonize their hosts and form biofilms. Here, different concentrations of phloretin were also tested for their capability to inhibit biofilm formation. At concentrations of 0.2 mM or 0.4 mM, phloretin significantly reduced biofilm formation by more than 60% (Figure 1C). Under the same level of growth inhibition (50% MIC) (Supplementary Figure 1A), ciprofloxacin, had no effect on biofilm formation, which was similar to that of the control treatments (Figure 1C).

### Effect of Phloretin on the Activity and Secretion of Plant Cell Wall-Degrading Enzymes (PCWDEs)

**FIGURE 2:**
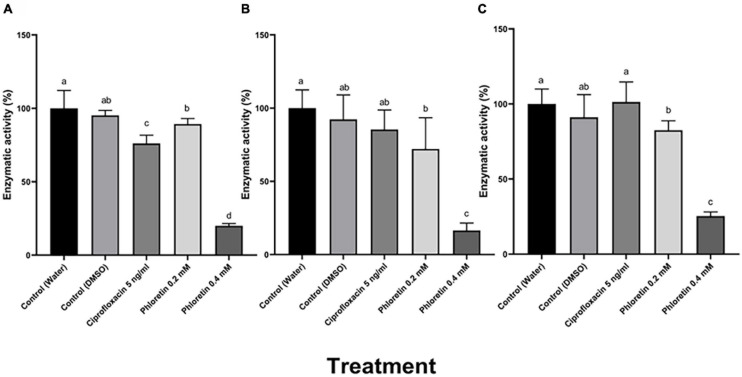
Effects of different concentrations of phloretin on exoenzyme activities in *Pectobacterium brasiliense* (Pb1692). **(A)** Pectate lyase (Pel) activity, **(B)** polygalacturonase (Peh) activity, **(C)** protease (Prt) activity. Extracellular enzyme activity was determined based on the halo area of substrate degradation. Results are expressed as the percentage of activity relative to controls (bacteria exposed to dH_2_O or 0.3% DMSO), bacteria were exposed to 0.2, 0.4 mM phloretin or ciprofloxacin, 5 ng/mL (50% growth inhibiting concentration). The Tukey-Kramer HSD test was used to analyze the differences. Bars that are not labeled with the same letter are significantly different from each other (*P* < 0.05; *n* = 16); Data represent means ± SE of 2 independent experiments, each with 8 replicates.

Plant cell wall-degrading enzymes (PCWDEs) including pectate lyase (Pel), polygalacturonase (Peh), protease (Prt) and cellulase (Cel), play an essential role in *Pectobacterium* virulence and cause soft-rot symptoms by degrading the host cell walls. A semi-quantitative method was used to assay the enzymatic activity (Figure 2; Chatterjee et al., 1995). Following 8 h of exposure to phloretin, dose-dependent reductions in the halo area were observed in the bacterial cultures that had been exposed to the phytochemical, representing a significant reduction in PCWDE activity. These levels of activity were assayed relative to a dH_2_O control and to 0.3% DMSO, which served as an additional control. The activities of Pel and Peh were reduced by more than 80%, while protease activity was reduced by more than 70% when Pb1692 was treated with 0.4 mM, as shown in Figure 2. Significant decrease in the production and secretion of PCWDEs was also observed at the lower phloretin concentration of 0.2 mM, a concentration that did not affect cell growth. Ciprofloxacin at 50% MIC (5 ng/mL), hardly affected Peh and Prt activities (Figures 2B,C), but inhibited Pel activity by 22% (Figure 2A).

### Effect of Phloretin on the Virulence of Pb1692 in Different Hosts

**FIGURE 3:**
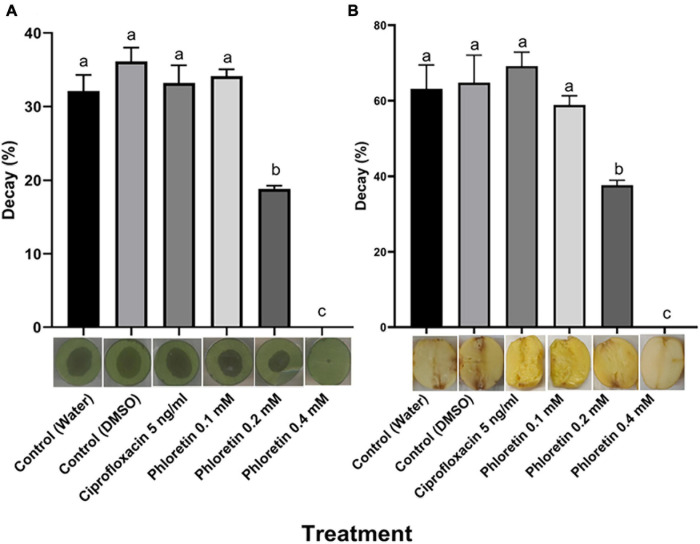
Effects of phloretin on *Pectobacterium brasiliense* (Pb1692) infection of **(A)** calla lily leaf discs and **(B)** potato tubers. Bacterial cultures of Pb1692 were exposed to phloretin or ciprofloxacin, 5 ng/mL (50% growth inhibiting concentration), for 2 h prior to the inoculation of the hosts. Bacterial suspensions were washed and pipetted onto calla lily or potato (10 μL, 10^6^ or 10^5^ CFU, respectively) and incubated at 28°C. The percentage of tissue showing signs of decay was determined at 15 h (calla lily) or 48 h (potato) post-inoculation, relative to a dH_2_O-treated control. DMSO (0.3%) was used as solvent and as an additional control. One-way ANOVA with *post hoc* Tukey-Kramer HSD tests was used to analyze differences. Bars represent the percentage of necrotic area with standard errors (*n* = 20 for calla lily; *n* = 8 for potato). Bars that are not labeled with the same letter are significantly different from each other (*P* < 0.05). Data represent one of two independent experiments with similar results. Representative photographs of infected calla lily leaf discs or potato tubers are provided for each treatment.

The significant reduction in PCWDE secretion following exposure to phloretin suggested that the ability of those bacteria to infect plant hosts might be impaired. To assess host infection following the exposure of *P. brasiliense* to phloretin, infection assays were conducted in two plant tissues, calla lily leaf discs and potato tubers, according to previously described infection protocols (Luzzatto et al., 2007; Joshi et al., 2015). The results revealed complete inhibition of infection after exposure to 0.4 mM phloretin, with no symptom development on the treated calla lily tissue (no decay), as compared to the decay observed in the dH_2_O and 0.3% DMSO control treatments (Figure 3A). A lower concentration of phloretin (0.2 mM) significantly inhibited infection on calla lily. Similar results were observed in potato tubers, where 0.4 mM phloretin completely inhibited infection while 0.2 mM significantly reduced it (Figure 3B). Disease symptoms in the presence of 5 ng/mL of ciprofloxacin (50% MIC) were not different from those of the control treatments, by this supporting the hypothesis that phloretin interfered with bacterial virulence (Figures 3A,B).

### Effect of Phloretin on AHL Biosynthesis in Pb1692

**FIGURE 4:**
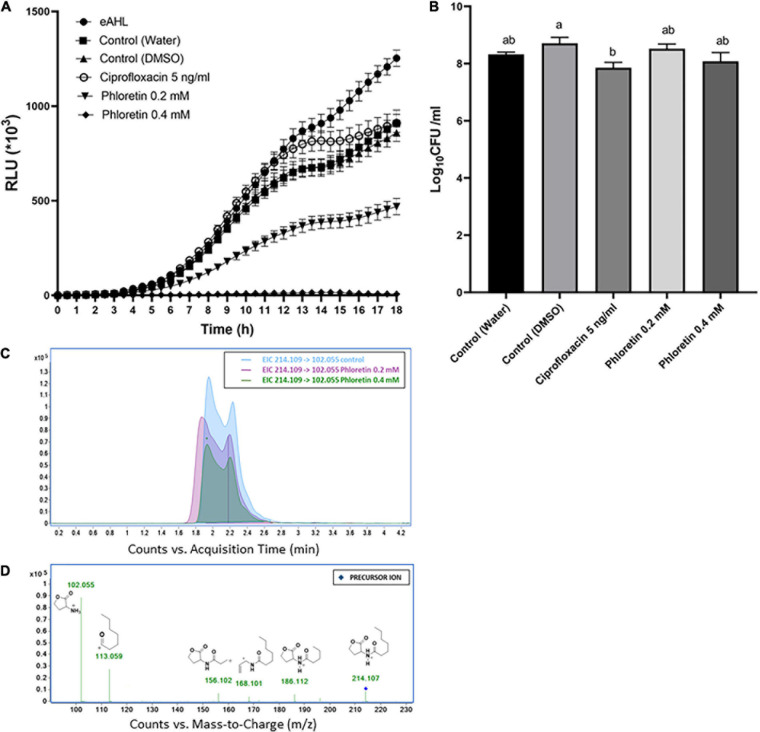
Effects of non-lethal concentrations of phloretin on the synthesis of AHL by *Pectobacterium brasiliense* (Pb1692). **(A)** Intensity of the luminescence produced by *Escherichia coli* pSB401 induced by eAHL and supernatants of Pb1692 grown in the presence of phloretin or ciprofloxacin 5 ng/mL, with dH_2_Oand 0.3% DMSO as controls. Luminescence (250 ms) and absorbance (600 nm) were measured every 30 min for 18 h, and the relative luminescence (RLU = LU/OD_600_) was calculated. Strain pSB401 supplemented with exogenous *N*-(β-ketocaproyl)-L-homoserine lactone (eAHL) at 100 nM served as a positive control. **(B)** Bacterial cells counted by serial dilution (CFU/mL) of Pb1692 cells grown in the presence of increasing concentrations of phloretin (dH_2_O control, 0.2 and 0.4 mM) or ciprofloxacin. Each data point represents the mean ± standard error (SE) of 8 replicates per treatment. Results from one of two independent experiments with similar results are presented. RLU – relative light units. One-way ANOVA with *post hoc* Tukey-Kramer HSD tests was used to analyze differences. Bars that are not labeled with the same letter are significantly different from each other. **(C)** Extracted ion chromatogram (EIC) of the protonated precursor ion [214.109, MH+]: 3-oxo-C6-HSL and its corresponding products ions. The x-axis represents retention time (min), and the y-axis represents signal intensity. **(D)** Fragmentation mass spectra by collision-induced dissociation (MS/MS) of 3-oxo-C6-HSL and metabolites. Precursor ions are indicated by in respective colors.

AHL molecules, mainly 3-oxo-C6-HSL and 3-oxo-C8-HSL, are the most common auto-inducer molecules to be produced by *Pectobacterium* strains in a cell density-dependent manner (Pollumaa et al., 2012; Valente et al., 2017). To gain insight into the effect of phloretin on the production of these auto-inducer molecules by Pb1692, two bacterial reporter strains were used. An *E. coli* strain pSB401 which detects and quantitatively reports the presence of AHL molecules that contain 6 to 8 carbons by producing a luminescence signal; and *Chromobacterium violaceum* (CV026) which can detect AHL molecules that contain 4 to 8 carbons, the presence of which induces the synthesis of a purple pigment, violacein. The intensity of the luminescence produced by pSB401 in response to phloretin is presented in Figure 4A. Supernatant of Pb1692 exposed to phloretin, at concentrations of 0–0.4 mM for 8 h, inhibited luminescence intensity in a manner proportional to the level of the signaling molecules that were produced by the bacterium. Exposure of Pb1692 to ciprofloxacin at 5 ng/mL (50% MIC), had no effect on AHL production, with RLU levels similar or higher than those of the control treatments (Figure 4A). In contrast, phloretin at 0.2 mM, a concentration that hardly affects growth, inhibited AHL production by 50%, while 0.4 mM completely blocked AHL production. An inverse relationship was observed between bioluminescence and phloretin concentrations (Supplementary Figure 4A). The analysis was performed relative to application of exogenous AHL (eAHL), dH_2_O-treated control and 0.3% DMSO, which was used as solvent for phloretin. At the end of the exposure to phloretin, bacterial cells were enumerated by dilution plating to analyze the number of living cells that contributed to the synthesis of AHL (Figure 4B). To rule out any effect of the compound on the biosensor strain pSB401, growth curves of the biosensor in the presence of the different treatments were produced, revealing no differences in the biosensor’s growth (Supplementary Figure 4B). In order to analyze the direct effect of phloretin on AHL production in Pb1692 following exposure to phloretin for 24 h, we have quantified 3-oxo-C6-HSL (Figures 4C,D) and 3-oxo-C8-HSL in the supernatant of the bacterium using LC-MS/MS. The results revealed more than five and ninefold reduction in 3-oxo-C6-HSL and 3-oxo-C8-HSL respectively (Supplementary Figures 4C,D), in response to 0.4 mM phloretin. In response to 0.2 mM phloretin a concentration that does not compromise growth, the reduction was also significant, allthough to a lesser extent.

**FIGURE 5:**
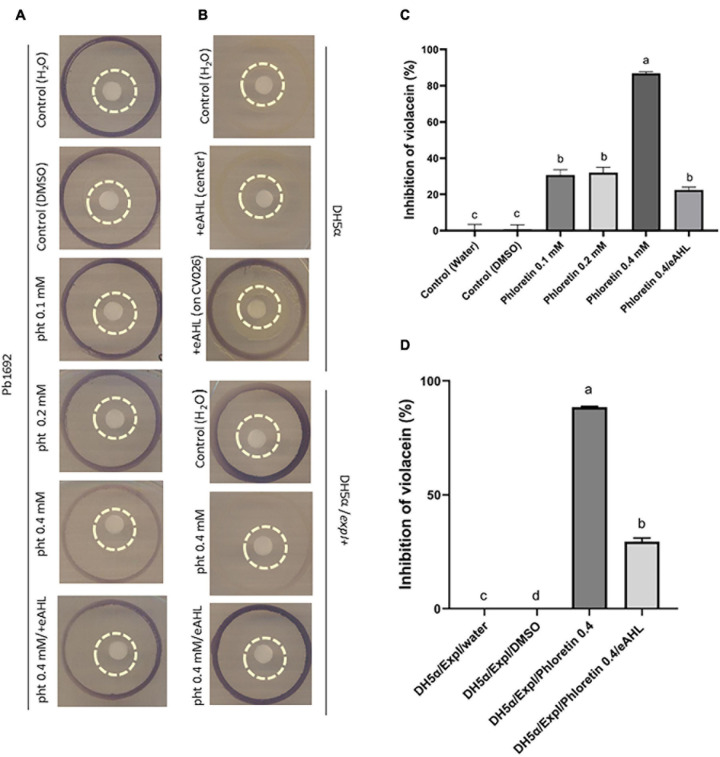
Effects of phloretin on AHL synthesis by *Pectobacterium brasiliense* (Pb1692) **(A,C)** and by *Escherichia coli* DH5α QS negative strain, complemented with *expI* from Pb1692 **(B,D)**. Visualization and evaluation of purple pigment (violacein) produced by CV026, in response to *N*-acyl-homoserine lactone (AHLs) produced by **(A)** Pb1692 and **(B)** transformed DH5α (pGEM), or DH5α complemented with *expI* (DH5α/pGEM *expI*). The bacteria were treated with 30 μL of dH_2_O or 0.3% (vol./vol.) of DMSO (control) or with phloretin. External AHL, eAHL, 30 μl of 500 nM of [*N*–(3–oxohexanoyl)–L–homoserine lactone (3-oxo-C_6_^–^ HSL) was applied to the central paper disc with phloretin 0.4 mM. The outer circle contained CV026, Pb1692 or DH5α/pGEM *expI* were at the inner circle, and treatments were applied on the central paper disc. [**(B)** upper section] QS-negative DH5α (pGEM), treated with 30 μL of dH_2_O or eAHL (500 nM) at the center of the paper disc. Positive control, 30 μL of eAHL (500 nM) added directly on the outer circle, to get the purple pigment. [**(B)** lower section] DH5α strain expressing AHL synthase of Pb1692 (pGEM *expI*) treated with 30 μl of dH_2_O or phloretin (0.4 mM). External AHL, *N*-(β-Ketocaproyl)-L-homoserine lactone (eAHL), 30 μl of 500 nM of AHL was applied to the central paper disc with 0.4 mM phloretin. **(C)** Quantitative determination of QS inhibition, shown as percent (%) of violacein inhibition by phloretin (0.1, 0.2, 0.4 mM, and 0.4 mM/eAHL), relative to dH_2_O or DMSO controls. **(D)** Quorum-sensing inhibition expressed as percent (%) violacein inhibition by phloretin (0.4 mM or 0.4 mM/eAHL) relative to the dH_2_O and DMSO controls. Data represent means ± SE of 4 replicates in 3 independent experiments. Bars that are not labeled with the same letter are significantly different from each other (*P* < 0.05).

The assays using CV026 were adapted and further modified from Hossain et al. (2017). An explanation and schematic describing the assay are provided in Supplementary Figure 5A. In short, the phytochemical, dH_2_O (control) or eAHL was applied to a paper disc (30 μL), which was placed on an LB agar plate, surrounded by an inner ring of Pb1692 and an outer ring of the reporter CV026 (Figure 5A). Inhibition of violacein pigment production by CV026 was quantified based on the ability of phloretin to inhibit Pb1692’s synthesis of AHL. The reporter strain, CV026, in the outer ring (surrounding Pb1692) turned purple upon sensing the signaling molecule (AHL). Phloretin (0.2 mM or 0.4 mM) was applied to the central paper disc from which it diffused to the surrounding bacterial inner circle of Pb1692. The diffusion of the higher concentration was enough to inhibit the synthesis of AHL by Pb1692, as demonstrated by the low production of violacein in the outer ring (Figure 5A). The application of exogenous AHL (eAHL) to the inner paper disc complemented violacein production by CV026 (Figure 5A). DH_2_O and DMSO (0.3%) served as controls. Since the former was a qualitative assay, the decrease in violacein production was quantified by a spectrophotometric assay that measured the absorbance of violacein at 585 nm and was expressed as percent inhibition relative to the control (Figure 5C and Supplementary Figure 5B). The growth of the reporter strain CV026, in the presence of the different supernatants (containing phloretin) showed that the treatments had no effect on growth of CV026, except for the 0.1 mM phloretin treatment, that slightly accelerated growth (Supplementary Figure 5F).

### Phloretin’s Effect on AHL Biosynthesis in DH5α

The QS-negative *E. coli* DH5α was used to explore a hypothetical direct interaction of phloretin with ExpI. To that end, the plasmid pGEM-*expI* was expressed in DH5α under the control of the T7 promoter (Joshi et al., 2020). The transformed strain (pGEM-*expI*) was able to produce AHL under control conditions, as shown by the strong synthesis of violacein by the reporter CV026 (Figure 5B, lower panel, top). The production of the pigment suggests that the signaling molecule was efficiently produced by DH5α pGEM-*expI*. Upon treatment with phloretin (0.4 mM, 30 μL), the synthesis of AHL by ExpI was almost completely blocked, as indicated by the absence of violacein pigment (Figure 5B, lower panel, center). At 0.4 mM phloretin had no inhibitory effect on DH5α growth (Supplementary Figure 5D), moreover, it was further diluted by the medium when applied to the paper disc, thus, the inhibitory effect was apparently the result of direct inhibition of ExpI, as no other components of the QS machinary are present in DH5α. Finally, eAHL was applied together with phloretin to the paper disc surrounded by DH5α pGEM-*expI*. The external application of the signaling compound recovered the presence of phloretin as observed by violacein production. To complete the observation, we made a quantitative determination of the inhibition caused by phloretin, relative to the dH_2_O control (%). That assay measured the production of violacein by CV026 in response to AHL produced by Pb1692 (Figure 5C) or DH5α pGEM-*expI* in the control treatment (dH_2_O), in the presence of 0.4 mM phloretin and in the presence of 0.4 mM phloretin and eAHL (Figure 5D and Supplementary Figure 5C). Growth of DH5α pGEM-*expI* was not compromised by phloretin (0.4 mM) as seen by growth curves kinetics and cell counts at the end of the analysis (Supplementary Figures 5D,E). Application of eAHL partialy recovered the inhibition caused by phloretin and complemented violacein production. However, while Pb1692 was able to recover the inhibitory effect of phloretin and once again produce AHL as observed by production of violacein by the reporter (Figure 5C), DH5α when tested at 37°C was unable to recover for violacein synthesis despite the addition of eAHL. When we conducted the experiment under similar conditions to Pb1692 (that is under 28°C), the production of violacein by the reporter was recovered, suggesting that the high temperature may affect AHL molecules and promote their degradation. To further explore the complementation effect by eAHL, we used *P. parmantieri* SCC3193 (WT) and its ExpI**^–^** mutant SCC3065 (*expI*^–^:Km^R^), which is unable to produce AHL (Pirhonen et al., 1991). CV026 was used as the biosensor for AHL production, which was tested upon application of phloretin, ciprofloxacin and eAHL (Supplementary Figure 6). While WT SCC3193 treated with 0.4 mM phloretin produced hardly any signaling molecules (weak violacein production by CV026), exogenous application of 3-oxo-C6-HSL (eAHL) together with phloretin complemented violacein production activity. Ciprofloxacin treatment (50% MIC), did not alter violacein production, suggesting that this level of growth inhibition, is not sufficient to notably inhibit AHL synthesis. The application of eAHL to the mutant SCC3065 (ExpI**^–^**) did not restore the biosensor’s production of violacein, suggesting that functional ExpI is required for the compensation. Finally, complementation of ExpI using expression vector (pET15b-*expI*) led to the full recovery of AHL production, as indicated by the accumulation of violacein. This suggests that ExpI is a target protein for inhibition by phloretin, but also suggests that it may be complemented by external signaling molecules (Supplementary Figure 6).

### Phloretin’s Effect on the Expression of Genes Associated With Virulence

**FIGURE 6:**
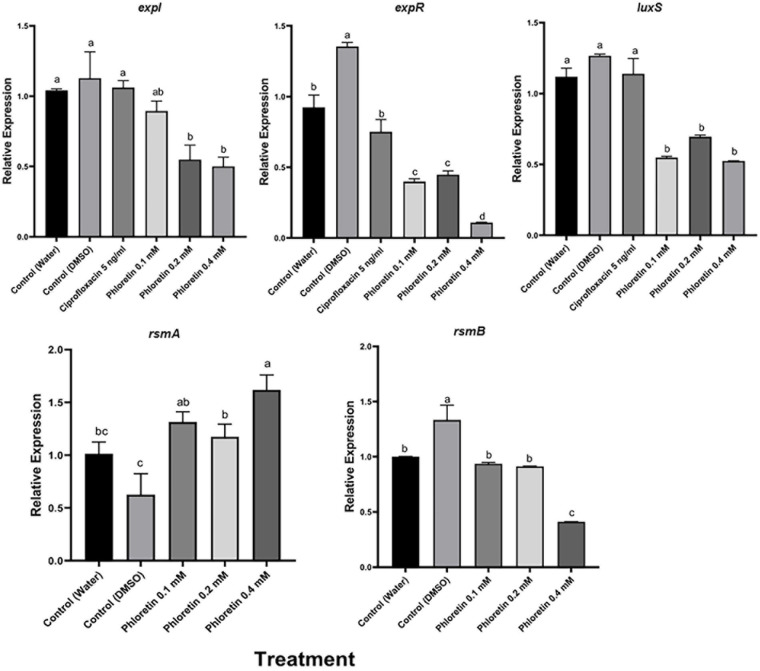
Effects of phloretin on transcript level of genes associated with quorum-sensing (QS) in *Pectobacterium brasiliense* Pb1692. The expression levels of the genes *expI*, *expR*, QS gene *luxS* (AI-2 system), *rsmA* and *rsmB* were determined by qPCR of cDNA samples prepared from RNA extracts of bacterial cultures grown in LB (28°C, 8 h), in the presence of phloretin (0.1, 0.2, and 0.4 mM), ciprofloxacin 5 ng/ml, dH_2_0 or 0.3% DMSO. Bars represent the relative gene expression observed in one experiment, means + standard errors (SE) of 6 replicates per treatment. The experiments were repeated twice. Bars that are not labeled with the same letter are significantly different from each other (*P* < 0.05; bar = mean + SE; *n* = 6).

We also examined the effects of phloretin on the relative expression levels of QS, motility and PCWDE-related virulence genes in Pb1692. The expression levels were evaluated by qPCR after the bacterial cultures had been exposed to phloretin or ciprofloxacin for 8 h. This time point was shown to be located in the logarithmic growth phase and suitable for the testing of expression levels of virulence genes (Joshi et al., 2016a). The tested genes were largely categorized as QS-system genes (*expI*, *expR*, *luxS*), QS regulators (*rsmA* and *rsmB*) and QS-regulated genes, mainly PCWDE (*pel*, *peh* and *prt*) and motility and biofilm-related genes that are linked to bacterial attachment and colonization of the host (*motA*, *fliA*, *fim*, *flhC* and *flhD*). At 0.4 mM, phloretin significantly suppressed the expression of QS-related genes, except that of the regulator *rsmA* (Figure 6). The expression of *expI* (AHL synthase) was suppressed twofold; whereas that of *expR* (response regulator) was suppressed 10- or 13-fold when compared with the dH_2_O and DMSO, control treatments, respectively (Figure 6). Surprisingly, DMSO (0.3%) increased QS-related activities under the experimental conditions. This may be due to the positive effect of a low dose of DMSO on Pb1692. The expression of *luxS* (AI-2 system) decreased 2- or 2.5-fold relative to the dH_2_O and DMSO treatments, respectively. Ciprofloxacin at 50% MIC did not alter the expression of *expI*, *expR*, or *luxS* and displayed a similar expression pattern as the control treatments. The relative expression of *rsmA* in response to phloretin increased relative to the dH_2_O and DMSO controls. The observed increase of *rsmA* expression was in line with the overall inhibition of QS machinery that we observed, as *rsmA* is a negative regulator of QS and the expression of PCWDE genes (*pel*, *peh* and *prt*) in pectobacteria (Cui et al., 1995). In view of that, relative expression of *pel* decreased 1.5-fold and the expression of *peh* and *prt* was repressed three and fourfold (respectively) relative to the dH_2_O treatment. As for the 0.3% DMSO treatment, the decrease in the expression of PCWDE genes was even more prominent, ranging from threefold for *pel* and up to sixfold for *prt* activity.

**FIGURE 7:**
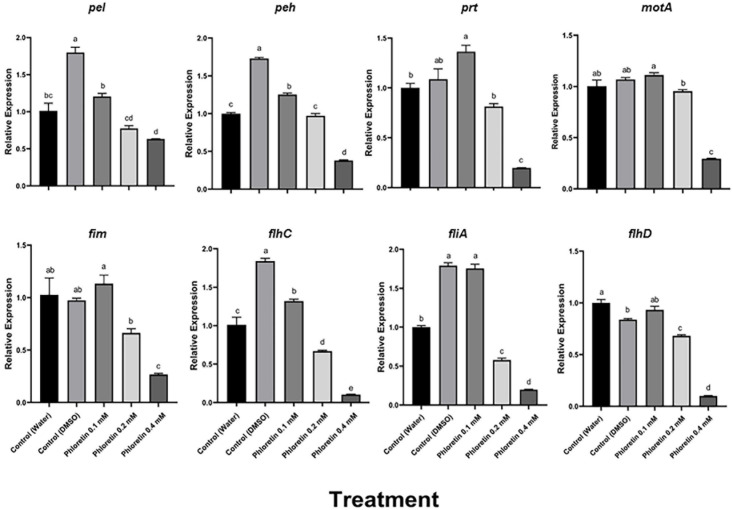
Effects of phloretin on transcript levels of genes associated with PCWDE and motility in *Pectobacterium brasiliense* Pb1692. The expression levels of PCWDE-related genes (*Pel, Peh* and *Prt*) and motility-related genes (*motA, fim, fliA, flhC* and *flhD*) were determined by qPCR of cDNA samples prepared from RNA extracts from bacterial cultures grown in LB (28°C, 8 h, continuous shaking at 150 rpm) in the presence of phloretin (0.1, 0.2, and 0.4 mM), dH_2_O or 0.3% DMSO. Bars represent the relative gene expression in one experiment, with means + standard errors (SE) of 6 replicates per treatment. The experiments were repeated twice with similar results. Bars that are not labeled with the same letter are significantly different from each other (*P* < 0.05; bar = mean + SE; *n* = 6).

Motility is another virulence factor that is at least partially regulated by QS (Josenhans and Suerbaum, 2002; Moleleki et al., 2017). Phloretin treatment also affected some motility-related genes, relative to the dH_2_O and 0.3% DMSO controls (Figure 7). Phloretin (0.4 mM) suppressed *motA* expression threefold; whereas *fliA* expression decreased fivefold relative to the dH_2_O control and 10-fold relative to the DMSO control. Similarly, *flhC* and *flhD* expression decreased about 10-fold in comparison to the dH_2_O control treatment of Pb1692, and the expression of *flhD* decreased even more relative to the DMSO control. Again, the expression of *flhC* and *flhA*, but not of *flhD*, increased in response to 0.3% DMSO, suggesting that the solvent had a positive effect on motility. The expression of *fim*, a gene responsible for attachment and biofilm formation, was repressed 2.5-fold, compared to both the dH_2_O control and the DMSO control.

### Molecular Docking of Phloretin to the ExpI Active Site

Molecular docking was performed to predict the potential interaction of phloretin with ExpI, the AHL synthase of *P. brasiliense*. A homology model for *P. brasiliense* ExpI was recently published (Joshi et al., 2016b, 2020). The Glide score value for the docking of phloretin to the active site of ExpI was −5.38 kcal/mol. The amino acids that were involved in the interaction were L32, W34, D45, Y47, M77, T81, F82, S100, R101, I142, V143, S144, M147, S166, E170 and V172.

**FIGURE 8:**
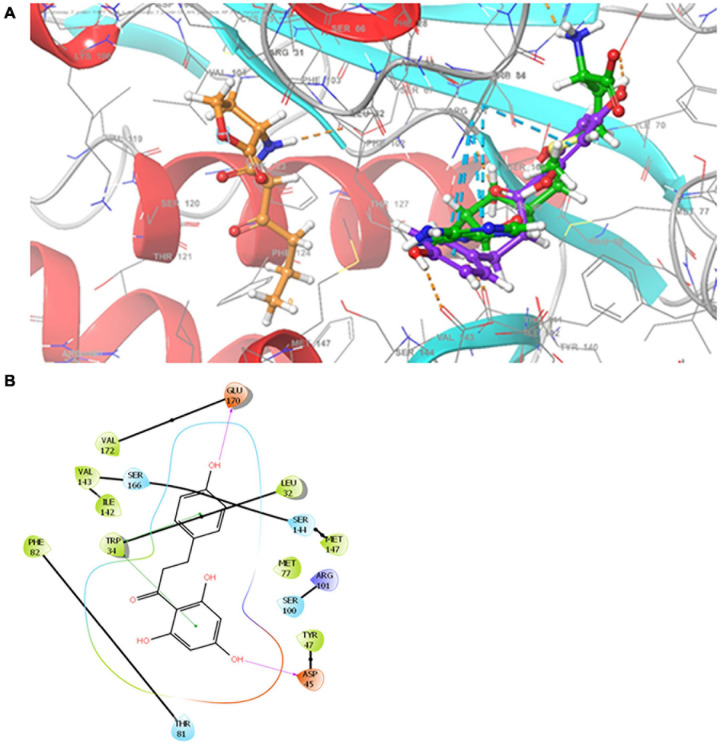
**(A)** Superimposition of 3-oxo-C_6_-HSL, SAM and phloretin. Binding modes for 3-oxo-C_6_-HSL, SAM and phloretin were determined by Glide XP at the binding site of ExpI. Carbon atoms of 3-oxo-C_6_-HSL, SAM and phloretin are colored orange, green and violet, respectively. Hydrogen bonds are presented as orange dashed lines and π−π stacking is shown as cyan dashed lines. **(B)** Two dimensional (2D) presentation of the interactions of phloretin in the binding site of ExpI (within 4 Å from the docked compound). Red, purple, cyan and green spheres represent positively charged, negatively charged, polar and hydrophobic residues respectively. Hydrogen bonds are presented as pink arrows and π−π stacking is shown as green line.

An improved homology model for ExpI was recently published based on TofI crystal structures of *Pantoea stewartii* EsaI and *Burkholderia glumae* (Joshi et al., 2020). This model was used here to depict the binding of phloretin to the active site of ExpI, which features two binding sites, one for the acyl chain of the acylated acyl-carrier protein and another for the substrate that is acylated by the action of SAM protein. Using the Glide program, allowed to reproduce the crystallographic poses to within 2.0 Å, the two sites were merged and used to examine the docking of phloretin in comparison to the previously described docking of 3-oxo-C6-HSL and SAM, the precursor molecule for AHL. The Glide score predicted values ranked 3-oxo-C6-HSL as the best binder (–6.4 kcal mol^–1^), followed by the precursor SAM (–6.2 kcal mol^–1^) and the phenolic compound phloretin (–5.4 kcal mol^–1^). Phloretin was shown to bind to the SAM cavity, with two hydrogen bonds with the backbone of Asp 45 and Glu170 and two π–π interactions with Trp 34 (Figures 8A,B). SAM binds to the SAM binding site, forming four hydrogen bonds with the backbone of Ile142 (two bonds) and the side chain of Glu43 and Arg101. SAM also participates in π–π stacking interactions with the side chain of Trp34 and makes two salt bridges with Asp45. Unlike phloretin, the QS molecule 3-oxo-C6-HSL binds to the acyl chain that is part of the merged binding site. It also has hydrophobic interactions and forms one hydrogen bond with the backbone of Phe102 (Joshi et al., 2020).

## Discussion

Intercellular communication that is based on quorum-sensing is activated when bacteria reach a certain population density. It is mediated by signaling molecules synthesized by an AHL synthase, of which acyl-homoserine lactones (AHLs) are the most common in Gram-negative bacteria (Gutierrez-Pacheco et al., 2019). We tested the capability of a plant-produced flavonoid – phloretin – to inhibit the QS system of Pb1692 and hypothesized a possible mechanism of action. Phloretin is produced by apple and pear, and in apple, it can confer resistance to *E. amylovora*, the pathogen that causes fire blight (Sklodowska et al., 2018). To study phloretin’s effect on Pb1692 virulence, we first calibrated a non-lethal concentration, in order to minimize the effect on bacterial growth. The MIC value on Pb1692 was consistent with previous reports on phloretin activity in *E. amylovora* strain 1189 (Ea1189; 250 μg/ml; 0.91 mM; Burse et al., 2004) and a later report (Al-Karablieh et al., 2009) on *E. coli* and Ea1189, that noted MIC values of more than 1,000 μg/ml (3.7 mM), an order of magnitude higher than the maximum concentration we used in our virulence assays. DMSO, which served as a solvent for phloretin, had a minor positive effect on bacterial cell growth, but had no effect on virulence. Similar stimulation of growth upon the use of DMSO was demonstrated in *Rhodobacter sphaeroides* (Horne and McEwan, 1998) and *Saccharomyces cerevisiae* (Hili et al., 1997). DMSO was demonstrated as a good solvent for aromatic organic compounds, which may use for toxicity studies of both individual bacterial strains and complex microbial assemblages (Modrzyński et al., 2019).

A study that involved the use of ciprofloxacin, an antibiotic that affects only cell division and growth, recently demonstrated that within a range of 50% growth inhibition, virulence traits such as PCWDE production and host infection by *P. brasilense* and *P. aroidearum* are hardly affected (Joshi et al., 2015). Since the efficacy of QS inhibitors was previously questioned mainly with compounds that also display toxicity to certain bacteria (Defoirdt et al., 2013), we included the antibiotic ciprofloxacin as an additional treatment. It allowed to test its efficacy as a virulence inhibitor under the same constrains for bacterial growth. Ciprofloxacin is a bactericidal agent, of the fluoroquinolone drug class, which prevents DNA replication by inhibiting bacterial DNA topoisomerase and DNA-gyrase, thereby inhibiting cell division (LeBel, 1988). The comparison of phloretin to ciprofloxacin under 50% MIC, demonstrated that phloretin at 0.4 mM, significantly impaired motility, biofilm formation, PCWDEs production, infection, and AHL synthesis, while ciprofloxcin hardly affected any of those traits. Moreover, many of the virulence associated phenotypes were also inhibited at 0.2 mM phloretin, a concentration that did not compromise bacterial growth. Phloretin has been reported to reduce motility and biofilm formation in several Gram-positive and Gram-negative bacteria (Zhou et al., 2015; Wang et al., 2017; Adil et al., 2019). Biofilms are complex three-dimensional structures of bacteria in a matrix consisting of exopolysaccharides (EPS), protein, lipids and extracellular DNA, attached to a biotic or abiotic solid surface (Castiblanco and Sundin, 2016). They protect bacteria from harsh environmental conditions and provide protection against antimicrobial compounds (Bogino et al., 2013; Castiblanco and Sundin, 2016). In the presence of non-lethal phloretin concentrations, biofilm formation in Pb1692 was significantly reduced, already at 0.2 mM phloretin, a concentration that had no effect on growth. A dose-dependent reduction in biofilm formation in response to phloretin was also reported in *E. coli* O157:H7, where it reduces cell aggregation and decreases biofilm adhesion (Lee et al., 2011; Wei et al., 2020). That inhibitory effect has been related to interference with auto-inducer (AI-2) QS and signaling (Lee et al., 2011). Regardless of these examples, most reports on biofilm inhibition by phloretin have involved Gram-positive bacteria such as *S. pyogenes*, in which 0.084 mM of phloretin not only inhibited biofilm formation, but also downregulated the expression of *srtB*, a gene involved in cell aggregation, as well as the expression of *luxS* and *dltA*, genes responsible for enhancing the hydrophobicity of the cell surface (Adil et al., 2019).

Motility is another important factor in pathogenesis of plant hosts. In *P. syringae* pv. *tomato*, phloretin affects bacterial fitness by inhibiting bacterial swimming and swarming motility, as well as T3SS expression (Vargas et al., 2013). In Pb1692, phloretin had significant effects on both swimming and swarming motility. The concentrations of phloretin used in our experiments were in agreement with those (0.3 mM) shown to affect *P. syringae* pv. *tomato* (Vargas et al., 2013). In *P. aeruginosa*, 0.1 mM phloretin was sufficient to reduce motility (Paczkowski et al., 2017). Phloretin also affects the expression of genes related to motility, including *fliA, fliC* and *fleQ*, as demonstrated by qRT-PCR (Vargas et al., 2013). The small reduction in expression levels of the sigma factor *fliA* and the sigma-dependent activator *fleQ*, significantly reduced the intercellular levels of FleQ and FliA proteins, which inhibited flagellin and flagella production, severely impairing swimming and swarming motility. The association between QS and motility in *P. brasilense* was recently demonstrated in an analysis of a Pb1692 mutant lacking *expI*, which displays lower swimming motility, showing an association between flagellar movement and QS regulation (Moleleki et al., 2017). Our findings regarding phloretin were in line with the findings showing that phloretin reduces swimming and swarming motility. At 0.4 mM, phloretin impaired swimming or swarming motility, while ciprofloxacin at 50% MIC had no effect on these traits. The results associated ExpI inhibition with attenuated motility. Analysis of the expression of motility-related genes in response to phloretin in Pb1692, revealed downregulation of flagellar motor (*motA*), flagellin (*fliA*) and flagellar transcriptional activator genes (*flhC* and *flhD)*. These genes have been recently shown to play roles in the motility of Pb1692, in an ExpI mutant of Pb1692 (Moleleki et al., 2017).

The effect of phloretin on AHL synthesis was assessed in Pb1692 using two common reporter strains. Violacein production in CV026 was significantly reduced following the exposure of Pb1692 to phloretin, as demonstrated in a qualitative and quantitative spectrometric assay. The second reporter strain pSB401, responds to nanomolar concentrations of 3-oxo-C6-HSL by generating luminescence that is proportional to the concentration of AHL (Winson et al., 1998; Teplitski et al., 2000; Middleton et al., 2002). Following the exposure of Pb1692 to phloretin, AHL production was decreased in a dose-dependent manner, as reflected by the reduced luminescence by pSB401. Ciprofloxacin at 50% MIC, had no effect on AHL production, and was similar or slightly above that of the control treatment. Interference with AHL synthesis occurred already at 0.2 mM phloretin a concentration that had no effect on bacterial growth, and to higher extent at 0.4 mM, where luminescence was completely inhibited. No effect of the compounds on the biosensor growth kinetics was observed under all treatments.

Finally, the inhibitory effect of phloretin on AHL synthesis in Pb1692 was demonstrated directly, using LC-MS/MS analysis for the detection. The results revealed a significant decrease of 3-oxo-C6-HSL and 3-oxo-C8-HSL in the supernatants of the bacterium, following exposure to the compounds.

To examine the hypothesis that ExpI is the main target of phloretin, the *expI* gene from Pb1692 was expressed in *E. coli* DH5α, a QS-negative strain that does not contain any component of the QS machinery. DH5α expressing the vector (pGEM) served as a negative control. As expected, this strain was unable to produce AHL. Expression of the *expI* gene in DH5α (pGEM-expI) under the T7 promoter resulted in AHL synthesis, as shown by the intense violacein production by CV026. Exposure of this strain to phloretin almost completely blocked AHL synthesis by ExpI (absence of violacein production by CV026). These results support the direct inhibition of ExpI by phloretin, a mechanism by which the compound interferes with the signaling molecule synthesis and, through that, the overall activity of the QS system in Pb1692.

The expression patterns of the QS genes *expI* and *expR* and the auto-inducer 2 (AI-2) system *luxS* were significantly downregulated in response to phloretin (0.1–0.4 mM concentrations), while ciprofloxacin had no effect on *expI*, *expR* or *luxS* expression. The transcription repressor *rsmA* was significantly upregulated, presenting an opposite trend to *rsmB*, which was also downregulated. The upregulation of *rsmA* and downregulation of *rsmB* upon phloretin treatment were not surprising, since RsmA is a negative regulator of PCWDE in the absence of AHL and the sRNA *rsmB* antagonizes its translational repression activities (Cui et al., 2005). The ExpI mutant of *P. parmentieri* exhibited more than threefold higher expression of *rsmA* in the absence of AHL, a pattern that was also observed upon the inhibition of ExpI by salicylic acid (Joshi et al., 2020). Higher levels of transcription of *rsmA* in pectobacteria have been strongly associated with the repression of virulence and reduced expression of PCWDE genes (Cui et al., 2005; Pollumaa et al., 2012; Valente et al., 2017). Accordingly, upon exposure to phloretin, the expression of *pel*, *peh* and *prt* was significantly downregulated, a reduction that was reflected in the overall attenuated virulence of the bacterium following exposure to phloretin. The secretion of PCWDEs is a virulence factor that is tightly controlled by QS (Barnard et al., 2007; Liu et al., 2008).

Infection assays were conducted in two common hosts, potato and calla lily. Upon treatment with phloretin, the bacterium was unable to initiate disease symptoms on either host. These results were in line with previous studies that have demonstrated the effects of several plant-derived phenolic acids and phenolic volatile compounds on the virulence of pectobacteria (Joshi et al., 2015, 2016a,b). They were also congruent with reports on the inhibition of virulence in *P. aeruginosa* by different flavonoids (Paczkowski et al., 2017). In the last decade, a number of publications have confirmed the involvement of plant extracts and more specifically, plant-derived phenolics including salicylic acid, carvacrol, eugenol, rosmarinic acid, curcumin and others in QS interference and the reduction of bacterial virulence (Adonizio et al., 2006; Annapoorani et al., 2012; Joshi et al., 2016a,b; Sivaranjani et al., 2016; Fernandez et al., 2018). As many natural compounds hold toxicity toward bacterial cells, it is not always straightforward to separate between different modes of action. In a recent excellent review (Defoirdt, 2018), QS inhibitors were defined as compounds that either inhibit or stimulate QS regulated gene expression, acting as stimulators or inhibitors of signal molecules biosynthesis, signal molecules detection, and interference with QS related phenotypes. Such inhibitors were specifically emphasized as valuable research tools, which may provide better insight with respect to the function of QS systems, which are unnoticed through gene knockout approach (Defoirdt, 2018). Moreover, inhibitors enable the analysis of QS interference and its broad spectrum effects on virulence, in a wide spectrum of strains and clinical isolates (Defoirdt, 2018). Here, the inhibitory effect of phloretin on QS, was supported by the absence of a similar effect upon application of the antibiotic ciprofloxacin under similar growth constrains.

Finally, to predict the potential interactions of phloretin with the active site of ExpI, a molecular docking analysis was conducted using a recently designed homology model of ExpI (Joshi et al., 2016b, 2020). The binding score of the protein–ligand complex was −5.38 kcal/mol, suggesting that phloretin does bind to the active site of ExpI. The predicted values were in the same range as those that have been calculated for the natural ligand 3-oxo-C6-HSL and the precursor SAM. In *P. aeruginosa*, phloretin has been shown to bind LasR and RhlR, the response regulators that positively regulate the expression of QS-related genes (Paczkowski et al., 2017). Although phloretin may also interact with ExpR, that interaction is not expected to affect virulence since, in pectobacteria, ExpR is a negative regulator and interference with its activity is not expected to reduce virulence.

In conclusion, phloretin interferes with virulence in *Pectobacterium* and is capable of interfering with QS-dependent signaling. Our results support the idea of using AHL synthase ExpI as a target for QS inhibition by natural compounds, in order to control soft rot bacteria. The inhibition of ExpI in the QS-negative DH5α by phloretin suggests that the compound directly binds ExpI, thereby inhibiting its downstream regulation of virulence. Further investigation is required to establish the overall effects of such plant-derived compounds on the whole range of gene expression in plant-pathogenic bacteria and dissect their exact binding site(s) and mode(s) of action.

## Materials and Methods

### Bacterial Strains, Growth Media and Chemicals

The bacterial strains used in this study are listed in Table 1. CV026 is a mini-Tn5 mutant of the wild-type strain that lacks AHL synthase and can only produce violacein when the AHL signal molecules is supplied externally (McClean et al., 1997; Kothari et al., 2017). The *E. coli* strain pSB401 does not produce AHL and contains a plasmid based on LuxR of *Vibrio fischeri* and *luxI* promoter controlling *luxCDABE* expression (Winson et al., 1998). Transformation of DH5α/*exp*I and SCC3065/*expI* was performed as described previously (Joshi et al., 2020). All strains were cultivated at 28°C in Luria-Bertani (LB) medium (Difco Laboratories, MI, United States). Strain pSB401 was cultivated at 37°C under continuous shaking at 150 rpm in a TU-400 incubator shaker (MRC, Holon, Israel). For the plant-inoculation assay, Murashige and Skoog minimal medium (MS; Duchefa, Haarlem, the Netherlands) was used. Solvents and chemicals were purchased from Sigma-Aldrich (Sigma-Aldrich), ciprofloxacin was purchased from Acros Organics. Phloretin was dissolved in DMSO to a concentration of 145 mM.

**TABLE 1:** Strains used in this study.

**Strain**	**Description**	**References/sources**
*Pectobacterium brasiliense* Pb1692	Dicot strain isolated from *Solanum tuberosum*, NCBI Accession no. PRJNA31121	Duarte et al., 2004; Glasner et al., 2008
*Chromobacterium violaceum* CV026	Mini-Tn5 mutant derived from *C. violaceum* ATCC 31532 HgR, cvil:Tn5 xylE, KanR, plus spontaneous StrR. AHL (C4–C8) biosensor, produces violacein (pigment) only in the presence of eAHL	McClean et al., 1997
*Escherichia coli* pSB401	luxRluxl’ (*Photobacterium fischeri* [ATCC 7744]):luxCDABE (*Photorhabdus luminescens* [ATCC 29999]) fusion; pACYC184-derived, TetR, AHL bioluminescent biosensor	Winson et al., 1998

### Determination of Minimum Inhibitory Concentrations

All compounds were purchased from Sigma-Aldrich (MO, United States). To determine the minimum inhibitory concentrations (MIC), the broth-dilution method was applied, as standardized by the Clinical Laboratory Standards Institute (CLSI 2012), with minor adjustments. Briefly, bacteria that had been grown for 16 h, 28°C in LB were normalized to 1 × 10*8* colony forming units (CFU) mL^–1^ with fresh liquid LB. Then, 190 μL of LB containing twofold serial dilutions of each of the tested compounds were inoculated with 10 μL of bacterial suspension containing 1 × 10*6* CFU in 96-well microtiter plates for 24 h, 28°C with continuous shaking at 150 rpm. The MIC was recorded as the lowest concentration of compound that was able to inhibit the visible growth of bacteria.

### Growth Curve

To study the growth curve, a 96-well microtiter plate was used. Bacterial cultures grown for 16 h, 28°C were diluted to a final concentration of 1 × 10*6* CFU in 200 μL of fresh LB containing non-lethal concentration of the compounds (or no compound, for the control) in a Tecan Spark^®^ multimode microplate reader (Tecan Trading AG, Switzerland) for 24 h. The plates were incubated at 28°C and cell density was measured at 600 nm every hour for 24 h, using a Tecan Spark^®^ multimode microplate reader (Tecan Trading AG, Switzerland). Each experiment was repeated at least twice with four replicates for each compound. Serial dilutions were made and plated onto LB agar plates for bacterial cell counting.

### Biofilm Formation

A microtiter dish assay with crystal violet (CV) was used to evaluate biofilm formation, as described by O’Toole (2011). Briefly, 200 μL of LB with or without the tested compound were inoculated with bacterial suspension containing 1 × 10*6* CFU in 96-well microtiter plate and incubated at 28°C for 72 h without shaking. The suspensions were discarded carefully and gently washed twice with double-distilled H_2_O (DDW) to remove any unattached cells. Then, 200 μL of 0.1% of CV was added to each well. The plate was incubated for 20 min at room temperature and CV from each well was discarded carefully. Each well was washed twice with DDW and the plate was dried before adding 200 μL of 30% acetic acid to solubilize the CV and incubated for 15 min at room temperature. The biofilm was quantified by measuring absorbance at 550 nm in a microplate reader (Spectra MR, Dynext Technologies, VA, United States).

### Enzymatic Assay

The production of plant cell wall-degrading enzymes (PCWDEs) was assessed semi-quantitatively, as described by Chatterjee et al. (1995). Briefly, plates were prepared as described and wells were made with a Number 2 cork-borer. The bacteria grown for 16 h, 28°C were put into fresh LB with or without a non-lethal concentration of one of the tested compounds with 1 × 10^6^ CFU for 8 h at 28°C under continuous shaking (150 rpm). Supernatant was transferred to a new Eppendorf tube after centrifuging at 14,000 rpm for 5 min. Each well of the plates was filled with 20 μL of supernatant and incubated at 28°C without shaking. After 18 h of incubation, pectate lyase (Pel) and polygalacturinase (Peh) plates were treated with 4N HCl and incubated for 15 m at room temperature. After 15 m clear haloes were developed around the wells. For the proteolytic enzyme (Prt) assay haloes developed after 24 h of incubation. Enzymatic activities were determined based on haloes area.

### Motility Assay

The swimming-motility assays were performed according to the method described by Sivaranjani et al. (2016). In the swimming assay, cultures grown for 16 h, 28°C, of the test bacterial pathogens were treated with phloretin (0, 0.1, 0.2, and 0.4 mM) or ciprofloxacin 5 ng/ml and stab-inoculated into the center of the medium containing 1% tryptone, 0.5% NaCl and 0.3% agar. The plates were then incubated for 24 h at 28°C without shaking and the distance that the bacterial cells migrated from the point of inoculation was measured. The swarming assay was performed according to method described by Koiv et al. (2013), with little modification. In brief, Pb1692 that had been treated with different concentrations of phloretin, ciprofloxacin or control and spotted onto minimal medium plates containing 0.4% agar and 10% yeast extract. The plates were then incubated in an undisturbed, upright position at 28°C for 72 h, after which the swarming migration zone was observed.

### AHL Detection Assays

#### *E. coli* pSB401 for Luminescence Production

For quantitative AHL detection, pSB401 was used as the reporter strain. This reporter contained the gene fusion luxRI’:luxCDABE, which responds to nanomolar concentrations of 3-oxo-C6-HS by generating luminescence that is proportional to the concentration of AHL. That luminescence was recorded using a luminometer (Winson et al., 1998). Bacterial strain Pb1692 was inoculated in 4 mL LB; whereas pSB401 was inoculated in 4 mL LB containing tetracycline (10 μg/mL). The two species were grown for 16 h at 28°C and 37°C, respectively, under continuous shaking (150 rpm). Pb1692 was centrifuged at 14,000 rpm for 5 min at room temperature and the supernatant was discarded. The bacterial pellet was re-suspended in fresh LB with or without a non-lethal concentration of the compounds and incubated at 28°C for 8 h with continuous shaking at 150 rpm. The reporter pSB401 was diluted 1:5 in fresh LB supplemented with 10 μg/mL tetracycline and incubated at 37°C. The control and treated suspensions of Pb1692 were centrifuged at 14,000 rpm for 5 min at room temperature and 20 μL of each supernatant were mixed with 180 μL of 5 × 10^6^ CFU/mL pSB401 in fresh LB medium in a 96-well microtiter plate. The reporter strain was used as blank and 50 nM of synthetic eAHL (Sigma, St. Louis, MO, United States) was used as the positive control. The plates were incubated at 37°C for 18 h in a Tecan Spark^®^ multimode microplate reader (Tecan Trading AG, Switzerland). Bioluminescence and optical density were simultaneously and automatically recorded every 30 min at 250 ms and 600 nm, respectively. To calculate bioluminescence, relative light units (RLU) were divided by optical density, as described by Winzer et al. (2000).

#### *Chromobacterium violaceum* for Violacein Production

For qualitative AHL detection, the CV026 strain was used. This strain produces the purple pigment violacein in the presence of AHL compounds with *N*-acyl C4-C8 side chains (McClean et al., 1997). A standard disc-diffusion assay was used to detect inhibition of AHL production by phloretin, using the procedure described by Hossain et al. (2017) with slight modifications. Pb1692 and CV026 bacteria that had been grown for 16 h, 28°C were washed by centrifuging at 14,000 rpm for 5 min and the supernatant was discarded. Then, both CV026 and Pb1692 were suspended in fresh LB at a concentration of 5 × 10^6^ CFU/mL. A small circular ring was made of Pb1692 and an outer, bigger ring was made with CV026; a paper disc was mounted in the middle of the inner ring. Thirty μL of each concentration of phloretin was gently poured onto the paper disc and dried for 30 min under a hood. Then, the plates were incubated at 28°C and the intensity of the purple pigment produced by the reporter strain was assessed.

The production of AHL was also quantified based on the ability of phloretin to inhibit the production of purple violacein pigment by CV026, as described by Hossain et al. (2017). Pb1692 at a concentration of 10^6^ CFU/mL was cultured in fresh LB containing different concentrations of phloretin (0.1, 0.2, and 0.4 mM) and incubated for 48 h at 28°C with continuous shaking (150 rpm). The culture was then centrifuged (5223 *g*, 10 min, 4°C) and the supernatant was transferred into a new Eppendorf tube. CV026 that had been grown for 16 h, 28°C was washed and suspended at a concentration of 5 × 10^6^ CFU/mL in fresh LB with an appropriate amount of antibiotic (kanamycin at 10 μg/mL). A total 500 μL of CV026 was placed with 500 μL of supernatant in a 1.7-mL Eppendorf tube and incubated for 24 h at 28°C with continuous shaking (150 rpm). The culture was then centrifuged (14,000 *g*, 10 min) to precipitate the insoluble violacein. The culture supernatant was discarded and the pellet was evenly re-suspended in 1 mL of dimethyl sulfoxide (DMSO). The solution was centrifuged (13,793 *g*, 10 min) to remove the cells and the violacein was quantified at a wavelength of 585 nm in a microplate reader (Spectra MR, Dynext Technologies, VA, United States).

The percentage of violacein inhibition was calculated using the following formula:

$$\%\text{violacein inhibition }=\;\frac{\text{OD of control at 585 nm }-\text{ OD of test sample at 585 nm}}{\text{OD of control at 585 nm}}\times 100$$

#### AHL Extraction

HPLC-MS/MS was used to quantify the AHLs produced by Pb1692 in the presence or absence of phloretin. The extraction of AHLs was performed as described previously, with minor modification (Ravn et al., 2001). Pb1692 was adjusted to approximately 5 × 10^6^ CFU/mL in LB supplemented with 0.2 or 0.4 mM of phloretin and cultured for 24 h, 28°C, at 150 rpm. After incubation, each culture was centrifuged at 8,000 *g* for 15 min, 4°C and the supernatant was transferred to a sterile tube. AHLs were extracted from the supernatant with ethyl acetate and 0.1% formic acid (v/v). The extraction process was repeated twice. Extracts were dried using SpeedVac vacuum concentrator (Savant SPD 111V, Thermo Scientific, MA, United States). The extract was resuspended in 20 μL of 99.5% acetonitrile (Alfa Aesar, United States) and analyzed by LC-MS/MS.

#### AHL Quantification

All the equipment for the analyses was from Agilent technologies (Santa Clara, CA, United States) and consisted of a 6545 QTOF mass spectrometer equipped with an electrospray ionization interface (ESI) coupled to a 1260 UHPLC, a G4204A quaternary pump, G4226A ALS auto-sampler, and G1316C thermostated column compartment. UHPLC was carried out on a ZORBAX RRHD Eclipse Plus C18, 95Å, 2.1 × 50 mm, 1.8 μm column, with water (0.1% formic acid)-MeCN gradient elution, from 5 to 95% MeCN for 10 min at a flow rate of 0.5 mL/min. Twenty μL of each sample were injected into the LC-MS/MS instrument in triplicates and an average peak area of three analyses was calculated. The water-MeCN solution was injected as a blank within a sequence of samples to confirm that there was no cumulative carryover. Mass spectral parameters were optimized for 3-oxo-C6-HSL by varying the fragmentor voltage of the ion source for scan mode and collision energy for product ion mode (MS/MS), 3-oxo-C8-HSL was quantified under the same parameters. Specific parameters of the ion source were readjusted. The ESI was operated in positive mode. The source temperature was set to 300°C, Nozzle voltage 500 V and ion spray voltage was 5.5 kV.

### Virulence Assay

To assess the effect of phloretin on the virulence of Pb1692, we measured the severity of symptoms in two plants, *Zantedeschia aethiopica* (calla lily) and *Solanum tuberosum* (potato), as described by Yishay et al. (2008). For this assay, fully expanded young leaves of calla lily and small (25–50 g) potato tubers were taken and surface-sterilized by soaking in 0.5% sodium hypochlorite for 20 min. Then, the samples were washed twice with sterile, double-distilled H_2_O and air-dried under a hood. For calla lily, leaf discs approximately 20 mm in diameter were excised and transferred to Petri dishes containing MS medium. For potato, whole disinfected tubers were used for the infection assay. Both the leaf discs and the potato tubers were pierced at the center with a sterile tip. Bacteria that had been grown for 16 h, 28°C in liquid LB were diluted to 1 × 10^8^ CFU/mL in sterile, double-distilled H_2_O with or without the test compound and incubated for 2 h at 28°C on a 150-rpm incubator shaker. After 2 h, leaf discs and potato tubers were inoculated with 10 μL of bacterial suspension (containing 10^6^ CFU) in the presence or absence of phloretin or ciprofloxacin. The inoculated plant material was incubated at 28°C without shaking. Disease severity in calla lily was expressed as the percentage of decayed tissues relative to the total disc area after 15 h. In potato, disease severity was assessed in terms of the percentage of tissue that had rotted after 48 h of exposure. The entire experiment was repeated at least twice with eight replicates for calla lily and four replicates for potato.

### RNA Extraction and cDNA Preparation

Pb1692 bacteria was grown for 16 h, 28°C in LB liquid medium under continuous shaking at 28°C. Then the fresh LB, with or without phloretin, was inoculated by transferring 2 μL of each bacterial strain in 4 mL liquid LB and incubating the bacteria for 8 h at 28°C under continuous shaking. After 8 h, a 2-mL sample was taken in an Eppendorf tube for RNA extraction. To extract RNA, the EZ-RNA II kit (Biological Industries, Kibbutz Beit Haemek, Israel) was used according to the manufacturer’s instruction. The extracted RNA was used to prepare cDNA using a cDNA synthesis kit (Applied Biosystems, Foster City, CA, United States). The cDNA reverse-transcription reaction was performed using a programmable thermal controller (MJ Research, St. Bruno, PQ, Canada) programmed to one cycle at 42°C for 30 min, followed by activation at 95°C for 2 min, after which the cDNA was stored at −20°C for future use.

### Quantification of mRNA by qRt-PCR

Real-time PCR was conducted to quantify the mRNA, as described by Joshi et al. (2016a). Briefly, all of the primers used in this study were designed using the National Center for Biotechnology Information (NCBI) primer BLAST software.^1^ The generated primers were 100–120 bp in size and the melting temperatures of the primers were designed for 60°C, with a difference of less than 5°C for each primer pair (Supplementary Table 1). To exclude the possibility of non-specific binding, primer sequences were analyzed by BLAST analysis (using NCBI BLAST software) against the database for the genus *Pectobacterium.* The primer mixture for qRT-PCR contained 5 μL of Fast SYBR Green Master Mix (Applied Biosystems) and 0.8 μL (5 μM) of each forward and reverse primer. Then, a total of 3.4 μL (17 ng) of cDNA was added to each well, so that the total reaction mixture would be 10 μL for each well. Reactions were performed using a Step One Plus Real-Time PCR system (Applied Biosystems) with the following cycling parameters: holding stage, 95°C for 20 s; cycling stage, 40 cycles of 95°C for 3 s and 60°C for 30 s; and melting curve stage, 95°C for 15 s, 60°C for 1 min and 95°C for 15 s. The data were analyzed by the comparative CT (ΔΔCT) method, with expression normalized to the expression of the reference gene *ffh*, as described by Takle et al. (2007).

### Molecular Docking

Homology modeling of ExpI and ExpR for *Pectobacterium* has been reported by Joshi et al. (2016b, 2020). We used the same ExpI homology model to examine molecular docking. The two binding sites were merged into a single site for the purpose of docking, so that the ligands were allowed maximal flexibility and were not constrained to the specific site. Docking of compounds was performed using a Glide XP procedure to the merged binding site of ExpI (Friesner et al., 2006). The ligand structures were drawn, converted into 3D coordinates and processed by Ligprep, to assign corrected protonation states at physiological pH.

### Data Analysis

Data were analyzed by one-way analysis of variance (ANOVA) using JMP Pro software (Version 14, Medmenham, Buckinghamshire, United Kingdom). When ANOVA indicated a significant difference (*P* < 0.05), a Tukey-Kramer multiple-comparison test was performed. Graphs were generated with GraphPad Prism Version 8.3.0 (GraphPad Software, San Diego, CA, United States).

## Data Availability Statement

The original contributions presented in the study are included in the article/Supplementary Material, further inquiries can be directed to the corresponding author/s.

## Author Contributions

MP, NK, OG, and MW conducted the experiments, analyzed the data, and prepared the manuscript. NK and HS analyzed computational docking data. MW analyzed LC-MS/MS data. IY and ZK planned and coordinated laboratory experiments. ZK, IY, and HS wrote and critically revised the manuscript.

## Conflict of Interest

The authors declare that the research was conducted in the absence of any commercial or financial relationships that could be construed as a potential conflict of interest.

## Footnotes

1 http://www.ncbi.nlm.nih.gov/tools/primer-blast/
