# Supplementary material for: Phloretin, an Apple Phytoalexin, Affects the Virulence and Fitness of Pectobacterium brasiliense by Interfering With Quorum-Sensing
Source: Front Plant Sci. 2021 Jun 25;12:671807. doi: 10.3389/fpls.2021.671807 (PMC8270676; doi:10.3389/fpls.2021.671807)
Supplement: Supplementary file 1 [file Data_Sheet_1.docx]

**Phloretin, an Apple Phytoalexin, Affects the Virulence and Fitness of *Pectobacterium brasiliense* by Interfering with Quorum-Sensing**

Manoj Pun¹י², Netaly Khazanov³, Ortal Galsurker¹, Zohar Kerem², Michal Weitman³, Hanoch Senderowitz³ and Iris Yedidia¹

**Supplementary Figures and tables:**

**Table S1.** List of primers used in the study.

| Primer Name | | Sequences | | | Use | | Origin |
| --- | --- | --- | --- | --- | --- | --- | --- |
| *expI_F* | TAC AAT AGC GGC AGG CAC TC | | QS signal generator; acyl-homoserine lactone synthase | | | Joshi et al. (2016a) | |
| *expI_R* | TGA GAA TCA GGA AGC ATT GGC | |  | | | Joshi et al. (2016a) | |
| *expR_F* | TGA GGT CAT GAG ATG TCG CC | | QS transcriptional regulator | | | Joshi et al. (2016a) | |
| *expR_R* | TTA TGC CGT CGT AGC GAT CC | |  | | | Joshi et al. (2016a) | |
| *LuxS_F* | CGG GGA TGG CGT GGA AAT TA | | S-Ribosylhomocysteinase; QS autoinducer-2 production | | | Joshi et al. (2016a) | |
| *LuxS_R* | GCT TTC CAG GAA TCG GCA AC | |  | | | Joshi et al. (2016a) | |
| *rsmA_F* | ATC GAT AAA TGT GTG GGG GA | | Controls the production of PCWDEs, carbon storage and cell motility | | | Joshi et al. (2016a) | |
| *rsmA_R* | TCA GGC CGA AAA ATC TCA AC | |  | | | Joshi et al. (2016a) | |
| *pel_F* | CTT CTT CAT GGC CGA TCC CA | | Secretion of pectate lyase | | | Joshi et al. (2016a) | |
| *pel_R* | CAA CGG ACT GTG GCT GAT TG | |  | | | Joshi et al. (2016a) | |
| *peh_F* | TAC CGC TAC GAG TAC GAC GA | | Secretion of polygalacturonase | | | Joshi et al. (2016a) | |
| *peh_R* | GAT CCC ACC AGC TCA CCT TT | |  | | | Joshi et al. (2016a) | |
| *ffh _F* | TGG CAA GCC AAT TAA ATT CC | | Signal recognition particle subunit (housekeeping gene) | | | Moleleki et al. (2017) | |
| *ffh _R* | TCC AGG AAG TCG GTC AAA TC | |  | | | Moleleki et al. (2017) | |
| *pro_F* | GCC GCA CCA AGT CAT TCT AT | | Putative protease | | | Moleleki et al. (2017) | |
| *pro_R* | CTT CCA GCG TTT CCA GTA GC | |  | | | Moleleki et al. (2017) | |
| *flhD _F* | TTC TGC GAT GTT TCG TCT TG | | Flagellar transcriptional activator FlhD | | | Moleleki et al. (2017) | |
| *flhD _R* | CGA CAA CAA AAT CCC CGT AT | | |  | | Moleleki et al. (2017) | |
| *fliA _F* | TAT CAA CAG CGC GAA AGA TG | | Flagellin protein | | | Moleleki et al. (2017) | |
| *fliA _R* | ATG CGC TGA GTC ATT TCC TT | |  | | | Moleleki et al. (2017) | |
| *fim_F* | GCT TTC ACA CTC GCC ATC TT | | Putative fimbrial protein | | | Moleleki et al. (2017) | |
| *fim_R* | GCC TTG CTC TCC GTA CTC AC | |  | | | Moleleki et al. (2017) | |
| *motA_F* | TTG CCT ACG GTT TTG TCT CC | | Flagellar motor; chemotaxis protein MotA | | | NCBI | |
| *motA_R* | ACA GCG TTT TAC GAC CGA AT | |  | | | NCBI | |
| *flhC_F* | ATT GCT GCA AAG GGA TGT TC | | Flagellar transcriptional activator FlhC | | | NCBI | |
| *flhC_R* | CCT GTT CAT CCA GCA GTT GA | |  | | | NCBI | |

**Figure S1**. Effect of of phloretin on growth of *Pectobacterium brasiliense* (Pb1692). (A) Growth curves of Pb1692 in the presence of increasing concentration of phloretin. DMSO (0.3%) was used as solvent and as an additional control to dH_2_O. Bacteria were grown at 28°C for 24 h and growth was assessed at 600 nm, every hour (bar = SE; n = 8). (B) Bacterial cell counts (Log 10 CFU mlˉ¹) of Pb1692 cells grown in the presence of increasing concentrations of phloretin for 24 h. Each bar represents mean ± standard error (SE) of 18 replicates per treatment. Bars that are not labelled with the same letter are signiﬁcantly different from each other (*P* < 0.05). One-way ANOVA with post hoc Tukey-Kramer HSD tests was used to analyze differences. Analysis was made by GraphPad Prism 8.0.

**Figure S2**. Swimming (A) and swarming (B) motility assays of *P. brasiliense* Pb1692 under exposure to increasing concentration of phloretin. Bacteria were grown at 28°C in M9 minimal media supplemented with 0.3% agar and tryptophan as carbon source for swimming and 0.5% agar and 10% Yeast extract for swarming.

**Figure S3**.  Bioﬁlm formation by *P. brasiliense* Pb1692 following exposure to increasing concentrations of phloretin. Bioﬁlm formation was assessed following 72 h of growth in the presence of phloretin in LB medium at 28°C. DH_2_O treatment or 0.3% DMSO were used as controls. Biofilm formed was stained by 0.1% crystal violet (CV) after gentle washing. Then, CV was rinsed by distilled water twice and dried at room temperature. The stained CV was then dissolved in 30% acetic acid.

**Figure S4**. Effects of non-lethal concentration of phloretin on AHL synthesis by *P. brasiliense* Pb1692. (A) Intensity of luminescence produced by *E. coli* pSB401 induced by supernatants of Pcb1692 grown with or without phloretin. Luminescence (250 ms) and absorbance (600 nm) were measured after 18 h, and relative luminescence (RLU = LU/OD 600 nm) was calculated. For positive control pSB401 was supplemented with exogenous N-(β-ketocaproyl)-L-homoserine lactone (eAHL) at 100 nM. DMSO (0.3%) or dH_2_O were used as controls. Each bar represents mean ± standard error (SE) of 6 replicates per treatment of one experiment, representative of two independent experiments. RLU, relative light units. (B) Growth curves of pSB401 following exposure to the different treatments for 18 h at 37⁰C (supernatants of Pb1692 grown in the presence of phloretin 0.2 and 0.4 mM, ciprofloxacin 5 ng/ml, or controls during 8h, 28^◦^C). Absorbance was measured every hour (OD 600 nm). (C, D) Peak areas of 3-oxo-C6-HSL (C) and 3-oxo-C8-HSL (D) from supernatants of Pb1692. Bacterial cells were culture in the presence of phloretin (0.2, 0.4 mM) or water (control), at 28ºC for 24 h. AHL was extracted from the supernatants and analyzed by LC-MS/MS 6545 QTOF mass spectrometer (Santa Clara, CA, USA). Bars represent the average of 2 independent experiments with 3 replicates each ± SE. Bars that are not labelled with the same letter are signiﬁcantly different from each other (*P* < 0.05). One-way ANOVA with post hoc Tukey-Kramer HSD tests was used to analyze differences. Analysis was made by GraphPad Prism 8.0.

**Figure S5**. (A) Schematic representation of qualitative agar diffusion assay for QS inhibition. (B, C) The effect of increasing concentrations of phloretin on AHL synthesis by *P. brasilense* Pb1692 or *E. coli* DH5α using the biosensor strain *C. violaceum* (CV026). (B) Photographs of violacein (purple pigment) produced by CV026, in response to *N*-acyl-homoserine lactones (AHL) synthesized by Pb1692; or (C) by transformed DH5α strain, complemented with *expI* gene from Pb1692 *(*DH5α /*expI*+). (D) Growth curves of DH5α in the presence of phloretin concentrations (0.1, 0.2, 0.4 mM) or control treatments, dH_2_O or DMSO. (E) Bacterial cell counts (Log 10 CFU mlˉ¹) of DH5α cells grown in the presence of the above concentrations of phloretin. Each bar represents mean ± standard error (SE) of 6 replicates per treatment. Bars that are not labelled with the same letter are signiﬁcantly different from each other (*P* < 0.05). One-way ANOVA with post hoc Tukey-Kramer HSD tests were used to analyze differences. Analysis was made by GraphPad Prism 8.0. (F) Growth curve of CV026 in the presence of the above treatments were diluted 1:1 v/v with 500 μL of 5 × 10⁶ CFU/mL of CV026 in fresh LB. Of which, 200 μL were used for the microtiter plate assay as described in the M&M section.

**Figure S6**. Effects of phloretin on AHL synthesis by *P. parmantieri* strains SCC3193 and its ExpI mutant strain SCC3065. Qualitative determination of purple pigment (violacein) produced by the reporter strain CV026, in response to phlorertin 0.4 mM, eAHL, or ciprofloxacin 5ng/ml applied to *P. parmantieri* SCC3193 (top) or SCC3065 complemented with *expI* from Pb1692 (bottom).
