# Supplementary figures and images for: Phloretin, an Apple Phytoalexin, Affects the Virulence and Fitness of Pectobacterium brasiliense by Interfering With Quorum-Sensing
Source: Front Plant Sci. 2021 Jun 25;12:671807. doi: 10.3389/fpls.2021.671807 (PMC8270676; doi:10.3389/fpls.2021.671807)

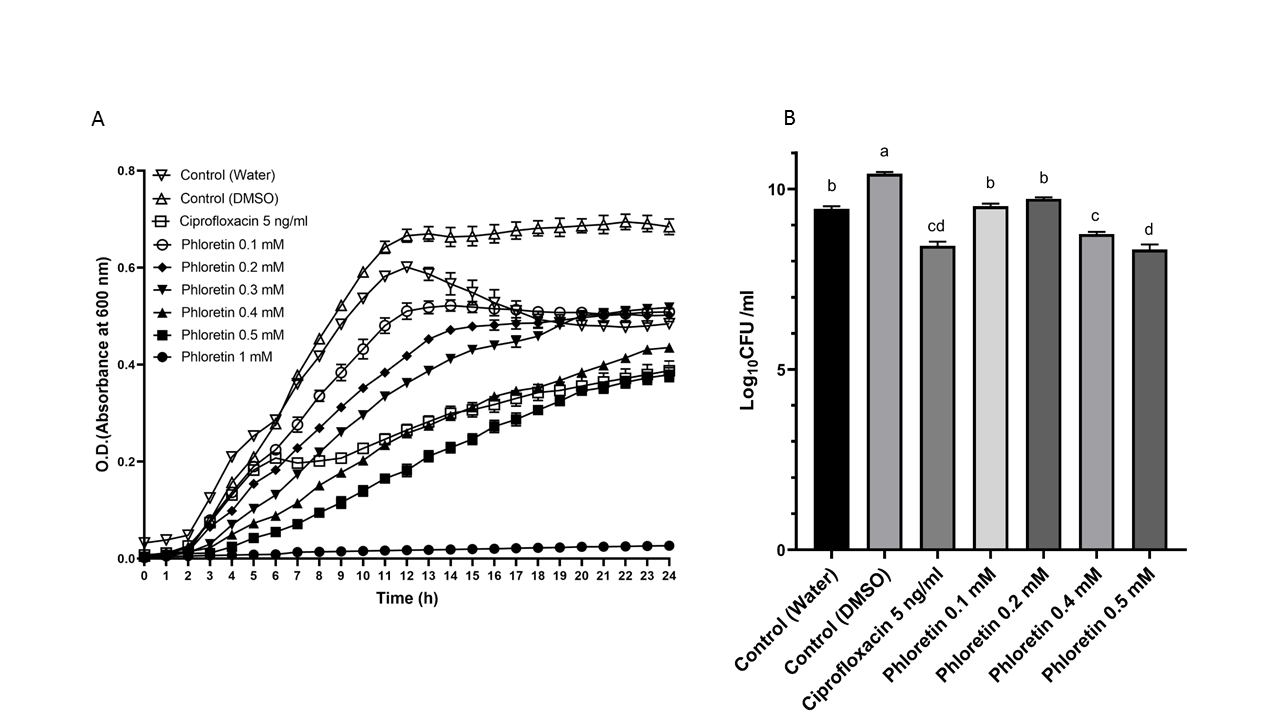

Supplement: Supplementary file 2 [file Image_1.TIF]

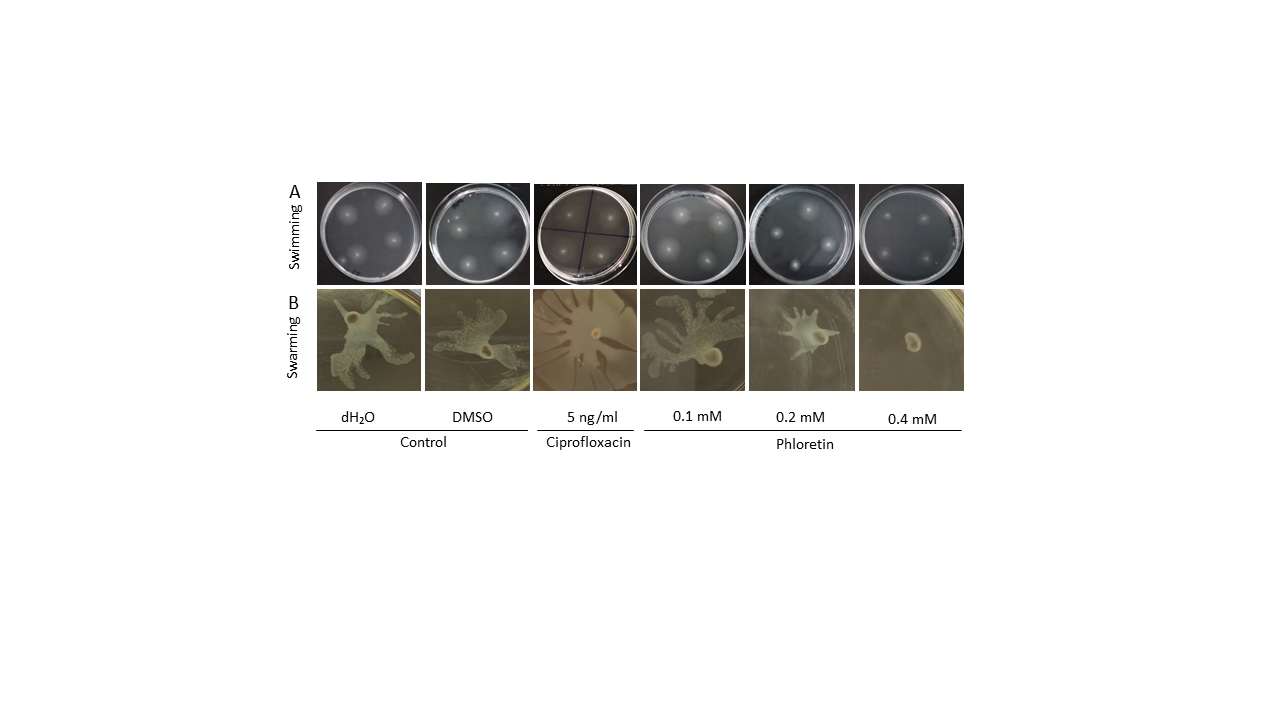

Supplement: Supplementary file 3 [file Image_2.TIF]

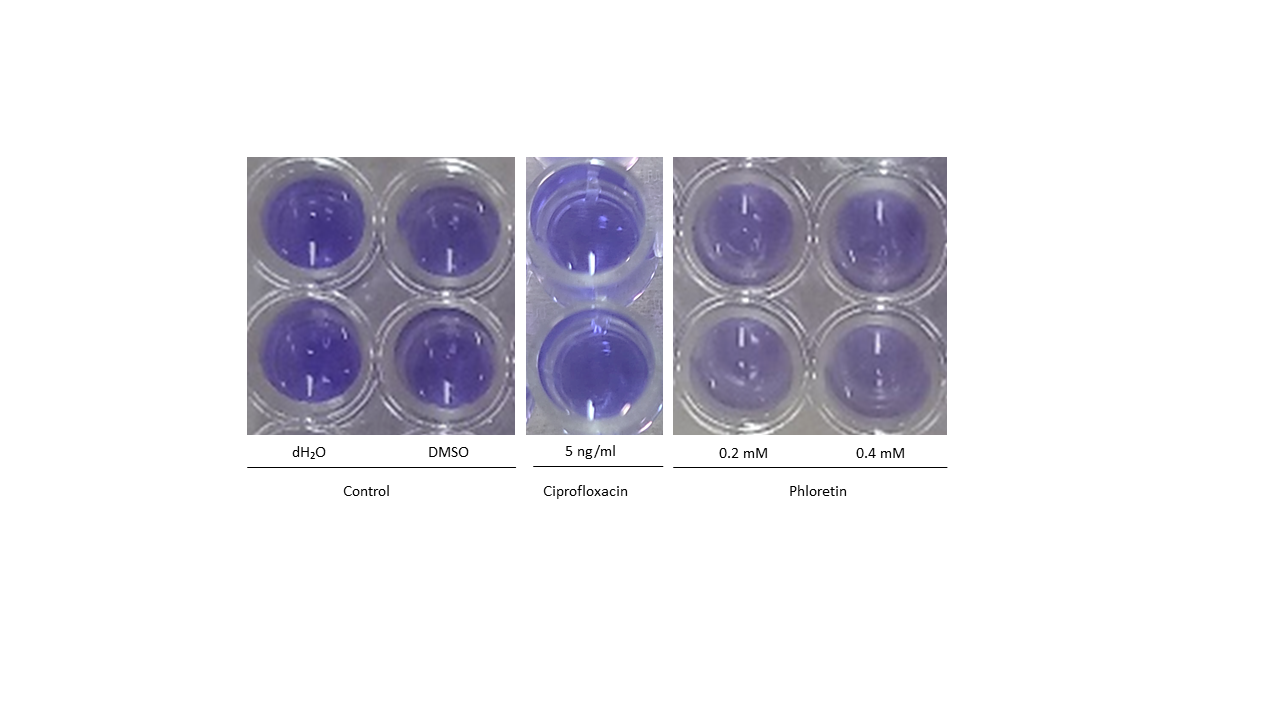

Supplement: Supplementary file 4 [file Image_3.TIF]

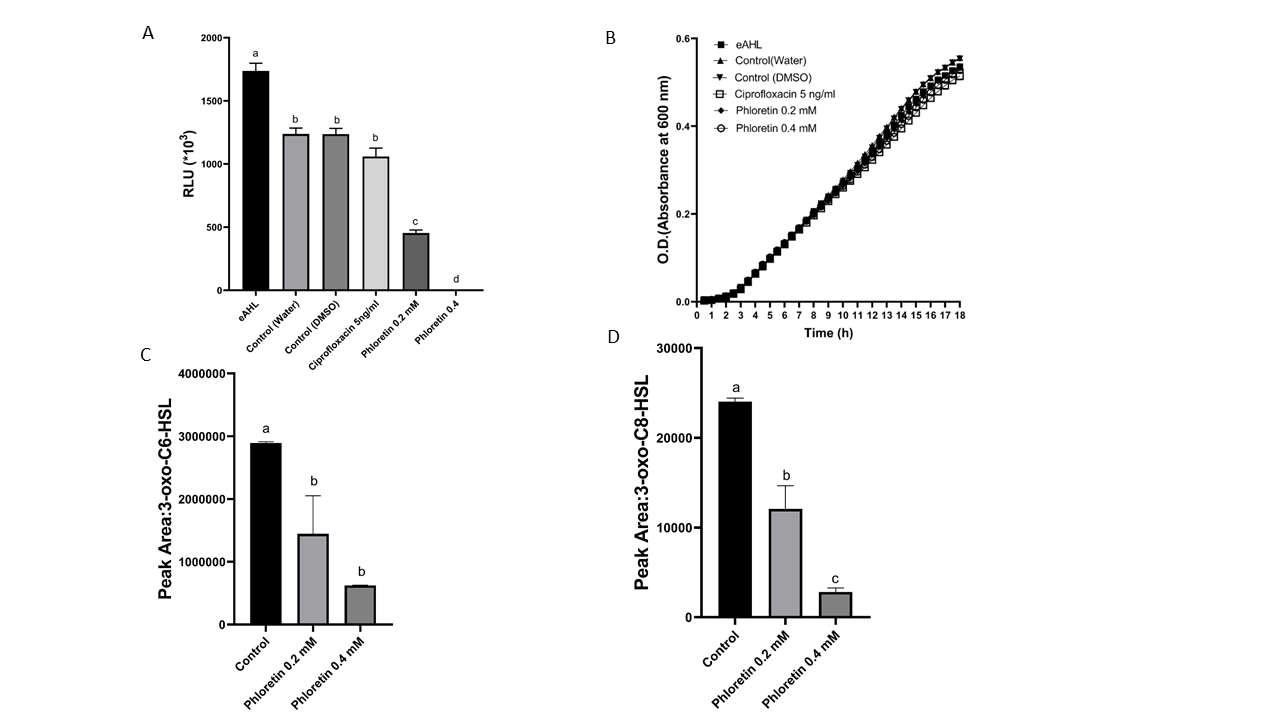

Supplement: Supplementary file 5 [file Image_4.TIF]

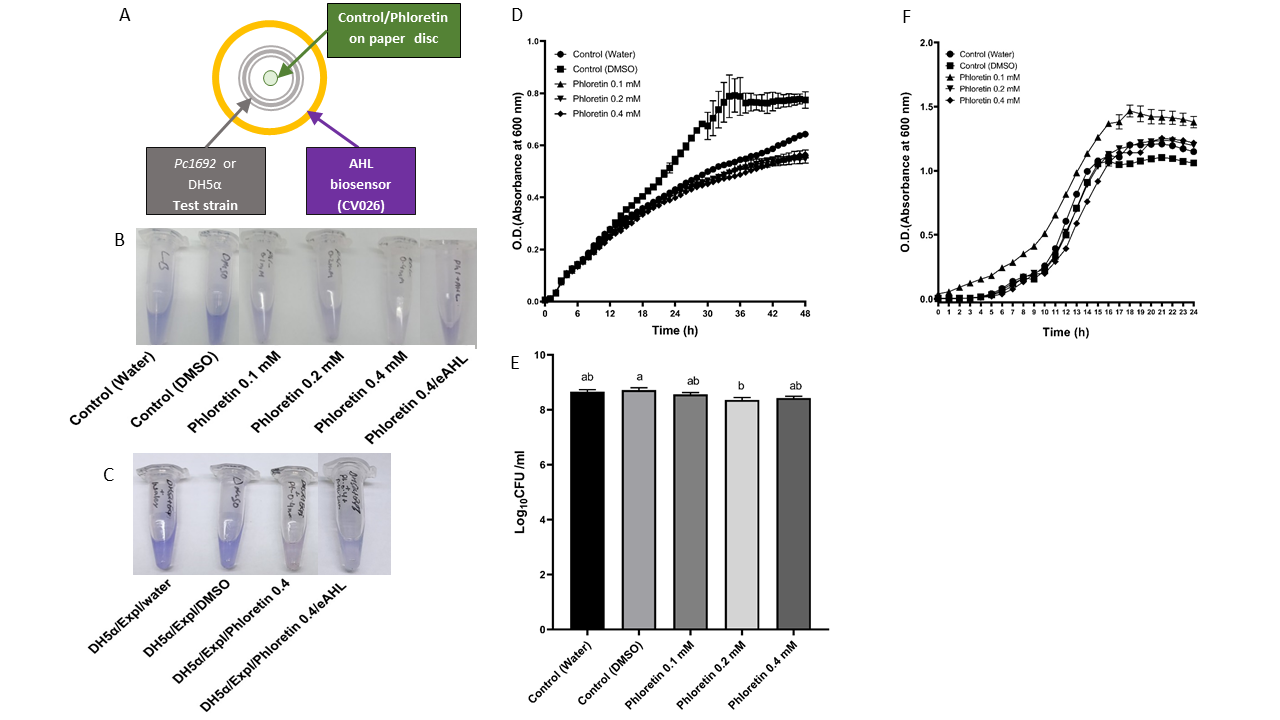

Supplement: Supplementary file 6 [file Image_5.TIF]

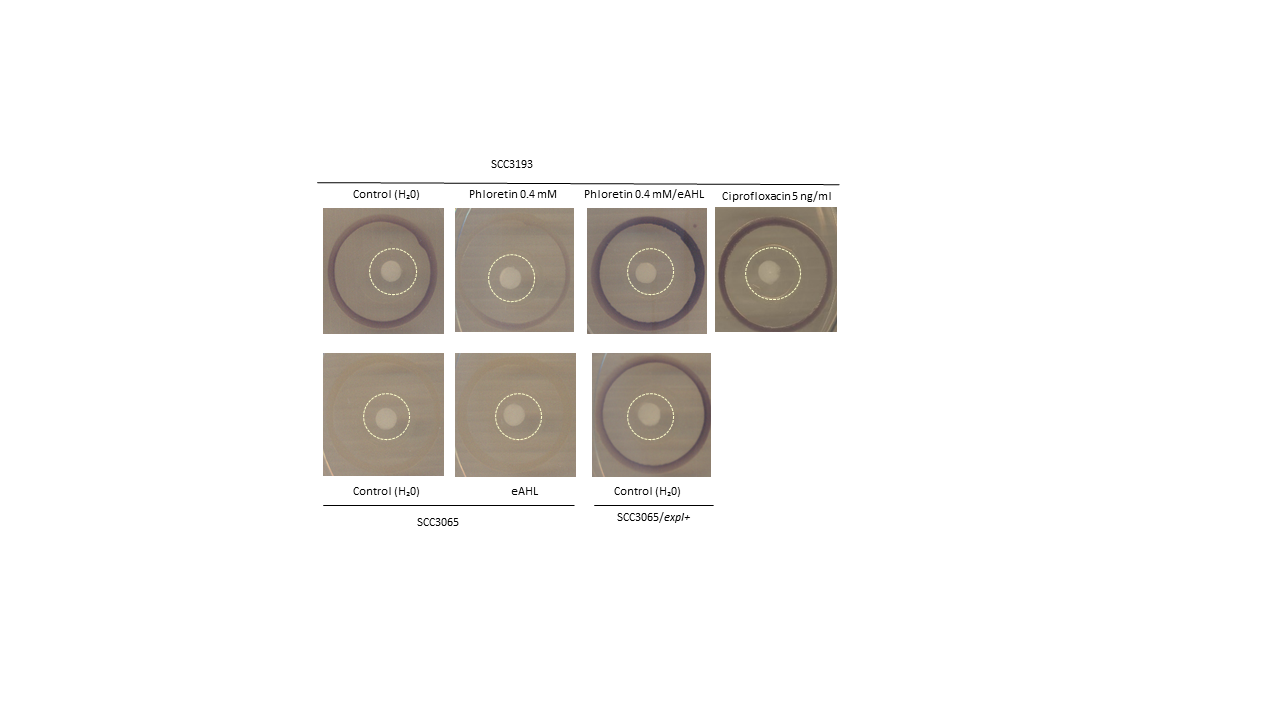

Supplement: Supplementary file 7 [file Image_6.TIF]
